# To plasticity and back again

**DOI:** 10.7554/eLife.06995

**Published:** 2015-03-12

**Authors:** H Frederik Nijhout

**Affiliations:** Department of Biology, Duke University, Durham, United Stateshfn@duke.edu

**Keywords:** developmental plasticity, complexity, nematodes, Pristionchus pacificus, evolutionary rates, *C. elegans*

## Abstract

Both the gain and the loss of flexibility in the development of phenotypes have led to an increased diversity of physical forms in nematode worms.

**Related research article** Susoy V, Ragsdale EJ, Kanzaki N, Sommer RJ. 2015. Rapid diversification associated with a macroevolutionary pulse of developmental plasticity. *eLife* **4**:e05463. doi: 10.7554/eLife.05463

**Image** Some worms develop a toothed mouth and others develop a small smooth mouth, depending on environmental cues

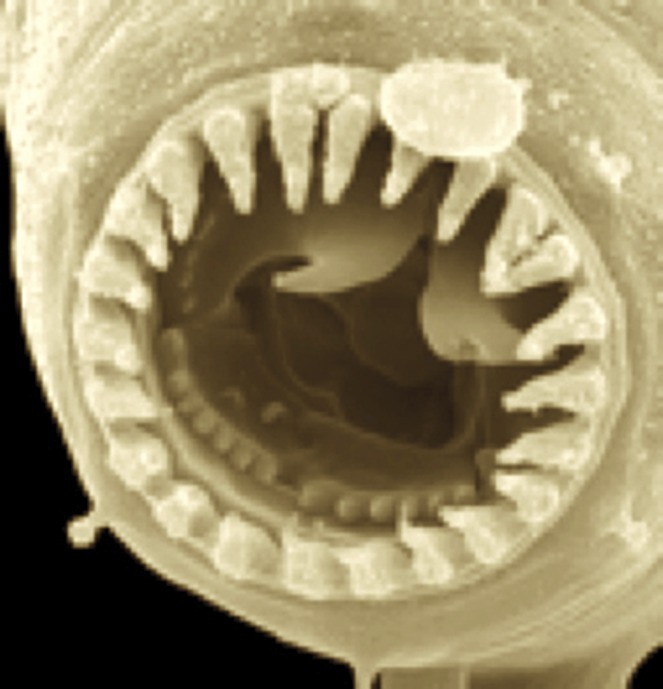

It was the Danish botanist Wilhelm Johannsen who coined the terms ‘genotype’ and ‘phenotype’. He thought that the genotype, or genetic makeup, of an organism referred to its ‘fundamental constitution’, whereas an organism's individual characters or traits, i.e. its phenotype, arose from a complicated interaction between the genotype and the environment (Johannsen, 1913).

Phenotypes can vary in response to external factors such as nutrition and temperature (Woltereck, 1909); this can be simply an accidental consequence of the fact that cellular and developmental processes that produce the phenotype run at different rates under different environmental conditions. And because environments are seldom, if ever, stable, it is very common for there to be some natural variation in phenotypes around some ‘ideal’ value, even between individuals with identical genotypes.

A natural question to ask is—does this variation matter? In terms of evolution, what matters are things that affect fitness: that is, things that affect the ability of an individual, compared to other individuals, to pass its genes to the next generation. If the phenotypic variation reduces fitness, then selection will favor the evolution of mechanisms that make the phenotype more robust, or less sensitive to environmental variation. On the other hand, if some of the phenotypes are well adapted to the environmental factors that induce them, evolution will favor mechanisms that stabilize the production of each phenotype in its best-suited environment.

When one genotype can produce more than one phenotype in different environments, this is generally referred to as ‘phenotypic plasticity’. As this ability can increase the diversity of phenotypes in a population, it is natural to ask whether populations of organisms with phenotypic plasticity are more able to evolve than those without. To date, there are various theories that suggest they should be, but there is little experimental evidence to support this view (Moczek et al., 2011; West-Eberhard, 2003). Now, in *eLife*, Ralf Sommer and Erik Ragsdale from the Max Planck Institute for Developmental Biology and co-workers—including Vladislav Susoy as first author—report that phenotypic plasticity is associated with evolutionary diversification in nematode worms (Susoy et al., 2015).

Susoy et al. examined a large group of nematode worms, including the model species *Caenorhabditis elegans* and some species with adults that have so-called ‘polyphenic’ mouthparts. Polyphenism is an extreme, but common, form of phenotypic plasticity in which two (or more) completely different phenotypes develop in response to specific environmental cues. Each of the alternative phenotypes is an adaptation to a different environment (Nijhout, 2003), and natural selection acts to ensure that they are stably and reliably produced to best match the environmental conditions.

Susoy et al. tested 90 species of nematodes for their ability to develop polyphenic mouthparts in the laboratory. In 23 species, some worms developed small smooth mouths specialized for feeding on bacteria, whereas others of the same species developed mouths with hooks and teeth. The toothed phenotype is used to feed on fungi and on other nematodes, and is induced by overcrowding and by starvation during the first larval stage (Bento et al., 2010; Sommer and Ogawa, 2011).

Analysis of the evolutionary relationships between the nematodes revealed that polyphenic mouthparts evolved only once in a common ancestor of the 23 species. Susoy et al. then asked if there was greater diversification in the shapes of mouthparts after polyphenism had evolved compared to the lineages of the ‘nematode family tree’ that had not evolved polyphenism. A sophisticated statistical analysis showed that this was indeed the case. This is some of the best empirical evidence to date for the theory that phenotypic plasticity enhances the ability of a population to evolve new forms (also known as evolvability).

Moreover, although polyphenism evolved only once, it appears to have been lost at least 10 times during the subsequent evolution of the lineage. Unexpectedly, the loss of polyphenism was followed by an even stronger rate of subsequent evolution of new traits and phenotypes. Thus the loss of phenotypic plasticity was also associated with an increase in evolvability. But how can this be explained?

One possible explanation is that a polyphenism requires developmental mechanisms that stabilize two alternative phenotypes, each in a different environment. If they work well, such stabilizing mechanisms buffer the two phenotypes against moderate changes to the organism's genetic makeup. This means that many mutations will not effect the phenotype and therefore will not be selected against. Such mutations will gradually accumulate in a population (Figure 1). Then, when the polyphenism is lost, the need to stabilize one of the two phenotypes disappears. Thus some of the accumulated genetic variation is no longer buffered and can cause the phenotype to vary more. This new phenotypic variation can now come under selection and lead to diverse adaptations in different lineages.

**Figure 1.**
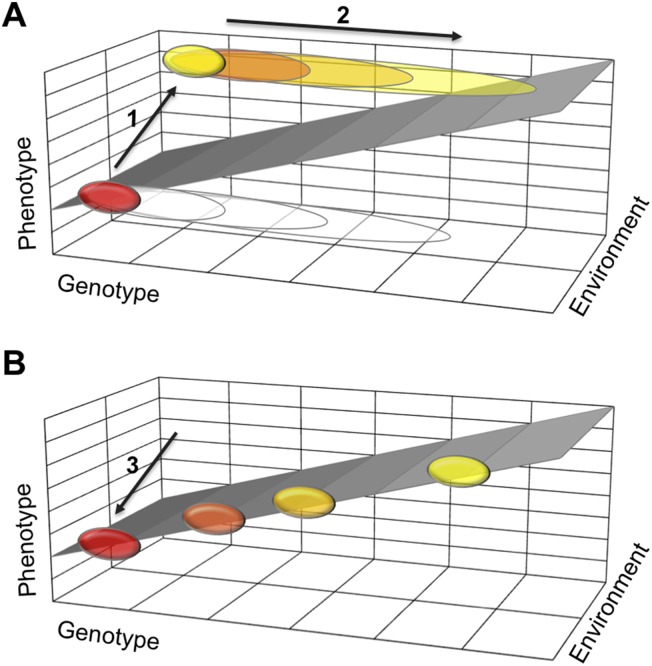
How a loss of phenotypic plasticity could increase evolvability. Changes in the genetic makeup, or genotype, of an organism can lead to changes in its traits and characteristics, also known as its phenotype. The sloped surface represents the hypothetical ideal relationship between genotype and phenotype in different environments, but in the absence of stabilizing mechanisms. (**A**) As part of a thought experiment, consider a population where at first all individuals have the same phenotype (shown as a red ellipse). This phenotype is stabilized by developmental mechanisms, which allow some genetic variation to accumulate (depicted as open ellipses expanding to the right). The evolution of a polyphenism (arrow 1) establishes a new phenotype (yellow ellipse) in a different environment, but with the same genotype. More genetic variation will accumulate (arrow 2) that has no effect on the phenotypes but improves stabilization of the alternative phenotypes in different environmental conditions. (**B**) When the polyphenism is lost (arrow 3), the mechanisms that stabilized the second phenotype are also lost. The accumulated genetic variation can now produce new phenotypes that (initially at least) fall on the ideal sloped surface, and that establish the foundation for the evolution of new traits and characteristics.

It is important to note that this explanation is, of course, a thought experiment that could be supported by statistical analyses. But the great challenge for the future will be to establish whether it is possible to devise experiments that can prove whether such a mechanism exists in nature.
